# Chikungunya in the Caribbean—Threat for Europe

**DOI:** 10.3201/eid2008.140650

**Published:** 2014-08

**Authors:** Jean-Michel Mansuy, Erick Grouteau, Catherine Mengelle, Isabelle Claudet, Jacques Izopet

**Affiliations:** Toulouse University Hospital, Toulouse, France (J.-M. Mansuy, E. Grouteau, C. Mengelle, I. Claudet, J. Izopet);; Institut National de la Santé et de la Recherche Médicale, Toulouse (J. Izopet);; Université Toulouse III Paul-Sabatier, Toulouse, (J. Izopet)

**Keywords:** chikungunya, chikungunya virus, viruses, outbreak, Caribbean, France, Europe

**To the Editor:** The first evidence of chikungunya virus in the Western Hemisphere was its detection in December 2013 in the French West Indies (*1*). One month later, the virus spread to other Caribbean islands.

Two cases of chikungunya in siblings (an 8-year old girl and a 10-year old boy) were recently identified at Toulouse University Hospital in southwestern France. Two days after these children had returned to France from the island of Martinique (French West Indies), acute fever associated with an arthromyalgic syndrome developed in these children. The children had maculopapular, nonpruriginous rashes on their arms and legs and endobuccal petechiae. The boy had bilateral knee effusions, and the girl had a measles-like rash that became more extended. Both children also had many mosquito bites that they scratched. They were discharged on the day of their admission. These 2 cases reported in metropolitan France after the patients visited Martinique indicate rapid spread of chikungunya virus.

We identified the virus by sequencing a 205-nt fragment within the envelope protein E1 gene of chikungunya virus (*2*) and performing phylogenetic analyses on the basis of reference sequences. This virus was a strain from Asia (Figure), whereas virus detected in 2 children in southeastern France in September 2010 had been imported from Rajasthan, India, and was an East/Central/South Africa strain (*3*). All of these strains did not show the single amino acid substitution in the envelope protein gene (E1-A226V) that favors adaptation for dissemination by *Aedes albopictus* mosquitoes (*4*) and would affect the potential magnitude of this outbreak.

**Figure:**
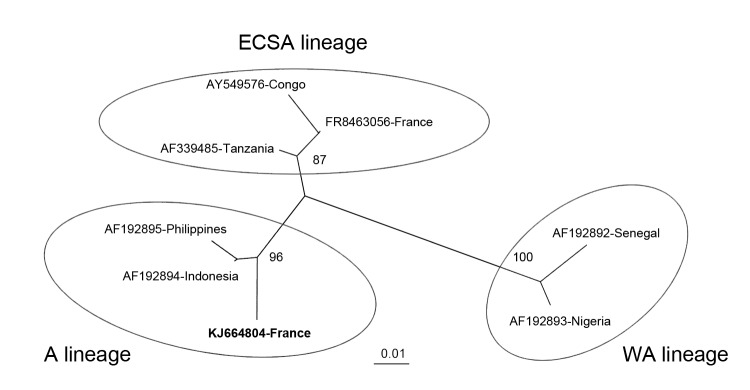
Phylogenetic tree constructed by using the neighbor-joining method and based on a partial (205 nt) sequence of the envelope protein 1 gene of chikungunya virus that was imported to metropolitan France from Martinique. Phylogenetic analysis includes reference sequences of chikungunya viruses from East/Central/South African (ECSA), West African (WA), and Asian (A) lineages. Sequences are indicated as GenBank accession number and country. The imported chikungunya strain isolated in this study is indicated in boldface. Bootstrap support values (100 replicates) are indicated at major nodes. Scale bar indicates nucleotide substitutions per site.

*Ae*. *aegypti* mosquitoes are common in the Western Hemisphere, where they are the major vector of urban dengue and yellow fever, and will facilitate spread of chikungunya in this region. *Ae*. *albopictus* (Asian tiger mosquito) is also an efficient vector of chikungunya virus and is found in many areas, including southern Europe. This mosquito species was responsible for the extensive chikungunya outbreak on La Réunion Island in the Indian Ocean (*5*) and was involved in the first chikungunya outbreak in Italy in 2007 (*6*). In these 2 outbreaks, human and mosquito virus strains contained mutation A226V in the envelope protein gene.

*Ae. albopictus* mosquitoes became established in a large area (91,150 km^2^) of southern France in 2013, where ≈13 million persons live. This mosquito, which is highly efficient in transmitting chikungunya virus (*7*), has been present in the study area for 2 years. For these reasons, a chikungunya/dengue national control program for continental France was established in 2006. The program involves rapid virologic diagnosis of imported or suspected autochthonous cases and vector control measures. This program operates during May–November, the period when *Ae. albopictus* mosquitoes circulate, and is based on entomologic surveillance data. The area covered by the program in 2013 was >10 times larger than that covered in 2006.

The presence of an effective vector, its progressive spread, and the outbreak of chikungunya in the Western Hemisphere increase concerns of a chikungunya outbreak in Europe (*8*). The greatest challenge is to find a way of interrupting the transmission chain of the virus as soon as possible. This challenge requires an effective policy of informing travelers at risk, early screening based on rapid virologic diagnosis, and effective vector control. Such control measures need an educated population to ensure emptying standing water from flowerpots, gutters, buckets, pool covers, pet water dishes, and discarded tires. They also need global antivector measures (eradication of eggs, larvae, and adults of *Aedes s*pp. mosquitoes).

These measures must be extremely efficient because an outbreak of chikungunya in the Western Hemisphere could spread rapidly. All countries in southern Europe are concerned by this public health challenge, and the battle against chikungunya requires rapid establishment of a supranational organization that should be able in real time to collect and return epidemiologic, virologic, and entomologic data. Although the usual movements of tourists around southern Europe during the summer will increase the number of persons at risk in this area, an even greater threat is the international movement of >600,000 persons expected to attend the next Soccer World Cup in Brazil in 2014 (*9*).
